# Supplementary material for: Nucleophagy is promoted by two autophagy receptors and inhibited by chromatin-nuclear envelope tethering in fission yeast
Source: Nat Commun. 2026 Mar 31;17:4678. doi: 10.1038/s41467-026-71237-x (PMC13201548; doi:10.1038/s41467-026-71237-x)
Supplement: Supplementary file 1 — Supplementary Information [file 41467_2026_71237_MOESM1_ESM.pdf]

Supplementary Information

Nucleophagy is promoted by two autophagy receptors and inhibited by chromatin-nuclear envelope tethering in fission yeast

Zhu-Hui Ma, Zhao-Qian Pan, Zhao-Di Jiang, Guang-Can Shao, Yu Hua, Fang Suo, Chen-Xi Zou, Yi-Feng Jiang, Meng-Qiu Dong, and Li-Lin Du

TABLE OF CONTENTS

Supplementary Figure 1 2
Supplementary Figure 2 3
Supplementary Figure 3 5
Supplementary Figure 4 7
Supplementary Figure 5 9
Supplementary Figure 6 11
Supplementary Figure 7 13
Supplementary Figure 8 15

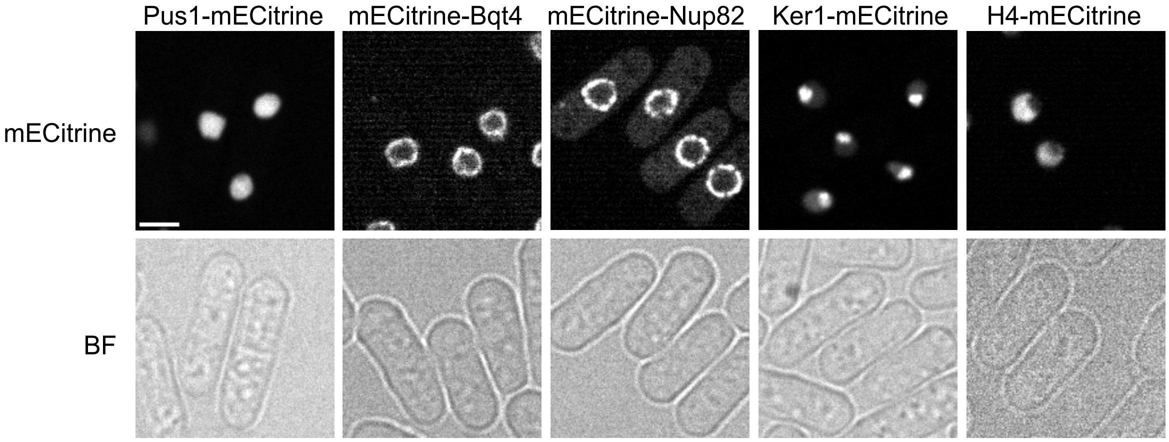

Supplementary Fig. 1: Subcellular localization of mECitrine-tagged nuclear proteins.
Log-phase cells expressing mECitrine-tagged nuclear proteins, including Pus1-mECitrine, mECitrine-Bqt4, mECitrine-Nup82, Ker1-mECitrine, and H4-mECitrine, from the *P41nmt1* promoter were examined by fluorescence microscopy. BF, bright field. Bar, 3 µm. The experiment was independently repeated three times with similar results.

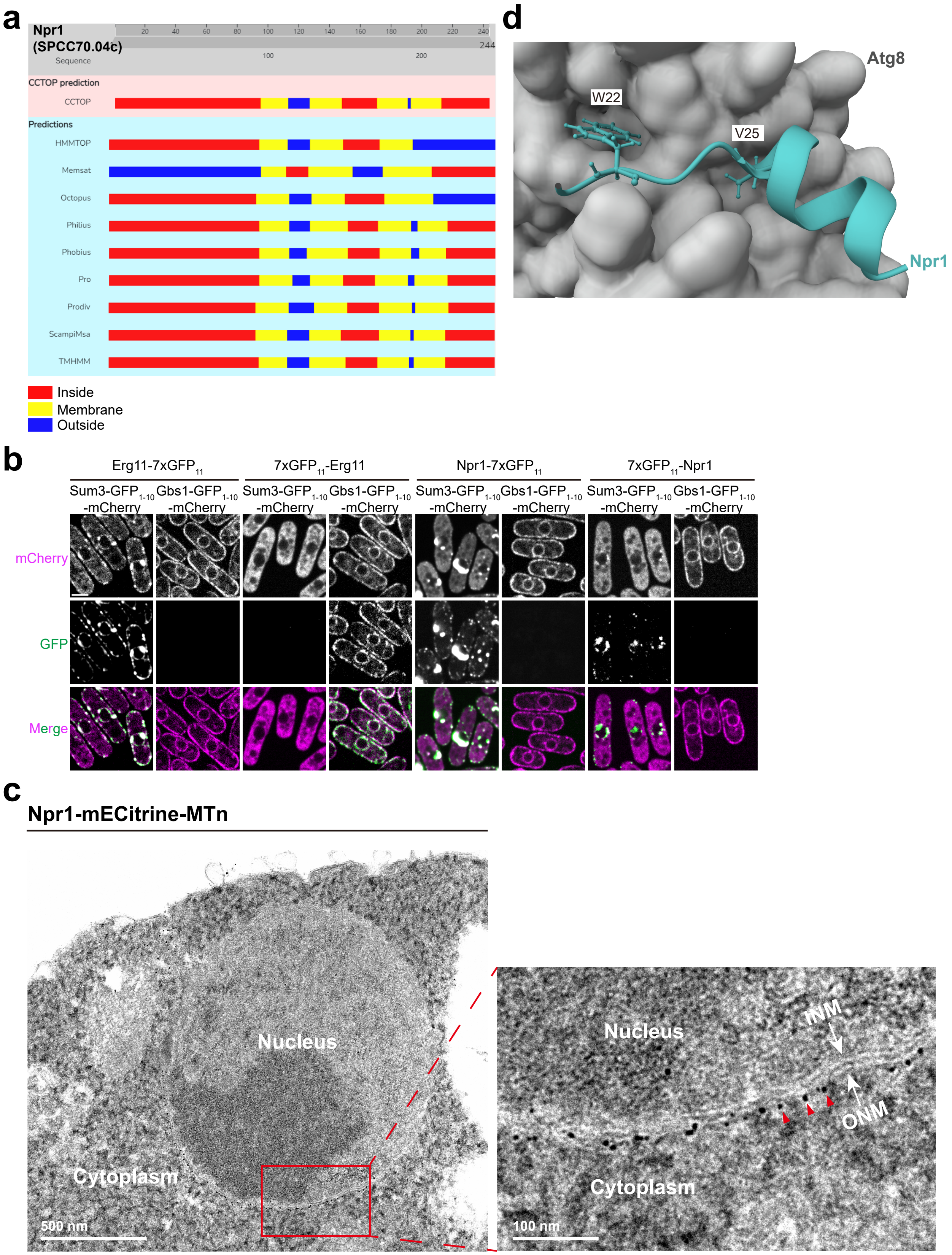

Supplementary Fig. 2: Identification of Npr1 as a candidate nucleophagy receptor.

(a) The complete results of predicting the membrane topology of Npr1 (SPCC70.04c) using CCTOP.
(b) Split GFP-based assays showed that the N- and C-termini of Npr1 are located in the cytosol. Log-phase cells were examined by fluorescence microscopy. $GFP_{1\text{-}10}$ fused proteins were expressed from the *adh1* promoter, and GFP_{11} fused proteins were expressed from the *P41nmt1* promoter. Bar, 3 μm. The experiment was independently repeated three times with similar results.
(c) Electron microscopy (EM) analysis of gold nanoparticle-labeled Npr1. MTn tagging of Npr1 allowed labeling with EM-visible gold nanoparticles. A magnified view of the boxed area is shown on the right. INM: inner nuclear membrane; ONM: outer nuclear membrane. Arrowheads indicate three representative gold nanoparticles. MTn-tagged Npr1 was expressed from the *Pnmt1* promoter. The experiment was independently repeated twice with similar results.
(d) The AlphaFold2-Multimer-predicted structure of the protein complex between Npr1 and Atg8. Amino acids 21-32 of Npr1 are shown in a ribbon representation, with W22 and V25 in the AIM highlighted in a ball-and-stick representation. Atg8 is shown in a surface representation and colored grey.

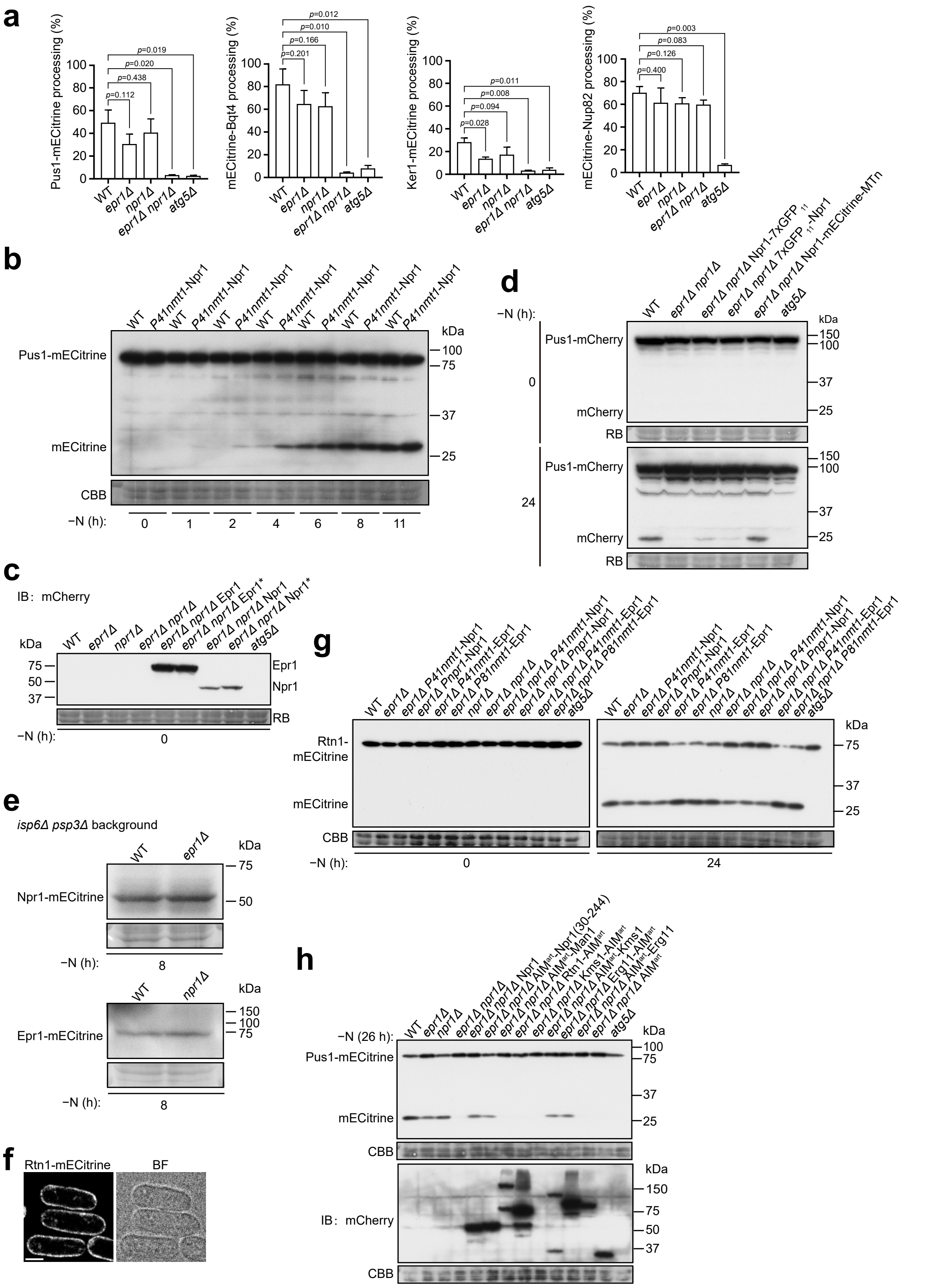

Supplementary Fig. 3: Npr1 and Epr1 are redundantly required for starvation-induced nucleophagy.

(a) Quantification of the percentages of Pus1-mECitrine, mECitrine-Bqt4, Ker1-mECitrine, and mECitrine-Nup82 processing after 24 h of nitrogen starvation in the experimental data shown in Fig. 3a. The quantification results are shown as the mean ± s.d. (n=3). The *p*-values were calculated using Welch's *t*-test.
(b) The overexpression of Npr1 through the addition of an extra copy of the *npr1* gene under the control of the *P41nmt1* promoter accelerated nucleophagy. The experiment was independently repeated three times with similar results.
(c) In the experiment shown in Fig. 3b, the expression levels of mCherry-tagged proteins expressed in *epr1Δ npr1Δ* were analyzed by immunoblotting using an anti-mCherry antibody.
(d) Ectopic expression of Npr1-mECitrine-MTn in *epr1Δ npr1Δ* cells rescued the nucleophagy defect of *epr1Δ npr1Δ* cells. Ectopic expression of Npr1-7×GFP_{11}, and Npr1-7×GFP_{11} in *epr1Δ npr1Δ* cells partially rescued the nucleophagy defect of *epr1Δ npr1Δ* cells. MTn-tagged Npr1 was expressed from the *P1nmt1* promoter. GFP_{11}-fused proteins were expressed from the *P41nmt1* promoter. The experiment was independently repeated twice with similar results.
(e) The loss of Epr1 did not impact the protein level of Npr1, and conversely, the loss of Npr1 did not affect the protein level of Epr1 after 8 hours of nitrogen starvation. *isp6Δ psp3Δ* background, which lacks vacuolar protease activities, was used to prevent the degradation of Npr1 and Epr1. Endogenously expressed Npr1-mECitrine and Epr1-mECitrine were analyzed by immunoblotting. The experiment was independently repeated twice with similar results.
(f) Subcellular localization of the cortical ER membrane protein Rtn1-mECitrine. Log-phase cells expressing Rtn1-mECitrine from the *P41nmt1* promoter were examined by fluorescence microscopy. Bar, 3 μm. The experiment was independently repeated twice with similar results.
(g) The deletion of *epr1* resulted in a moderate defect in the autophagic degradation of the cortical ER membrane protein Rtn1 during nitrogen starvation, and this phenotype was not further exacerbated by the additional deletion of *npr1*. The experiment was independently repeated three times with similar results.
(h) Fusion of the artificial AIM (AIM^{art}) to Man1, Rtn1, the lumen-facing C-terminus of Kms1, or the lumen-facing N-terminus of Erg11 did not rescue the nucleophagy defect of *epr1Δ npr1Δ* (Top). The expression levels of the AIM-fused proteins tagged with mCherry were analyzed by immunoblotting using an anti-mCherry antibody (Bottom). The experiment was independently repeated three times with similar results.

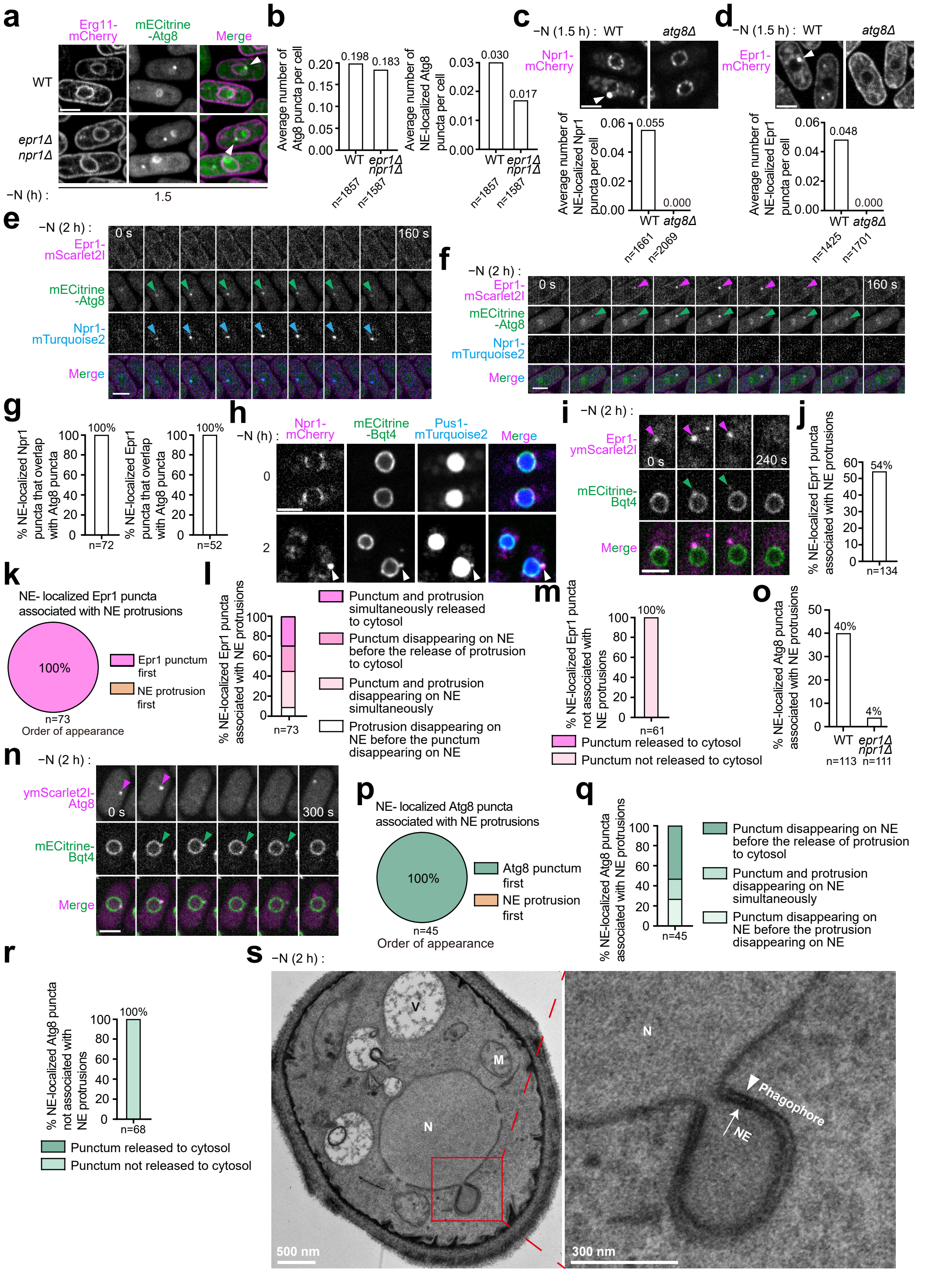

Supplementary Fig. 4: Epr1 and Npr1 puncta overlap with some but not all NE-localized Atg8 puncta, and NE protrusions are released into the cytosol.

(a) NE-localized Atg8 puncta (arrowheads) in WT and *epr1Δ npr1Δ* cells after 1.5 h of nitrogen starvation. Erg11 marks the ER membrane. Bar, 3 μm. The experiment was independently repeated three times with similar results.
(b) Average total and NE-localized Atg8 puncta per cell from (a).
(c) NE-localized Npr1 puncta (arrowheads) were absent in *atg8Δ* cells after 1.5 h of nitrogen starvation. Top: images. Bottom: quantification.
(d) As in (c), for Epr1.
(e–f) Time-lapse of cells with a type II (e) or type III (f) punctum. Bar, 3 μm.
(g) Percentages of NE-localized Npr1 (left) and Epr1 (right) puncta that exhibited overlap with Atg8 during the lifespan of a punctum from appearance to disappearance. Cells co-expressing Epr1-mScarlet2I, mECitrine-Atg8, and Npr1-mTurquoise2 were imaged at 20-s intervals after 2 h of nitrogen starvation.
(h) Pus1-mTurquoise2 localized at Npr1 puncta-associated NE protrusions. Bar, 3 μm.
(i) Time-lapse showing a Bqt4-labeled NE protrusion with an Epr1 punctum released into the cytosol. Cells co-expressing Epr1-ymScarlet2I and mECitrine-Bqt4; 80-s intervals after 2 h of nitrogen starvation. Bar, 3 μm.
(j) Percentage of NE-localized Epr1 puncta with NE protrusions (2 h starvation).
(k) Order of appearance of Epr1 puncta and associated NE protrusions.
(l) Percentages of NE-localized Epr1 puncta and associated NE protrusions released into the cytosol or disappearing from the NE.
(m) Epr1 puncta lacking associated NE protrusions were not released into the cytosol.
(n) An Atg8 punctum disappeared before its associated NE protrusion was released. Cells co-expressing ymScarlet2I-Atg8 and mECitrine-Bqt4; 60-s intervals after 2 h of nitrogen starvation. Bar, 3 μm.
(o) Percentage of NE-localized Atg8 puncta with NE protrusions in WT and *epr1Δ npr1Δ* cells (2 h starvation).
(p) Order of appearance of Atg8 puncta and associated NE protrusions.
(q) Fate of NE-localized Atg8 puncta and associated NE protrusions.
(r) NE-localized Atg8 puncta not associated with NE protrusions were not released into the cytosol.
(s) Electron microscopy showing a phagophore wrapping around an NE protrusion (WT, 2 h starvation).

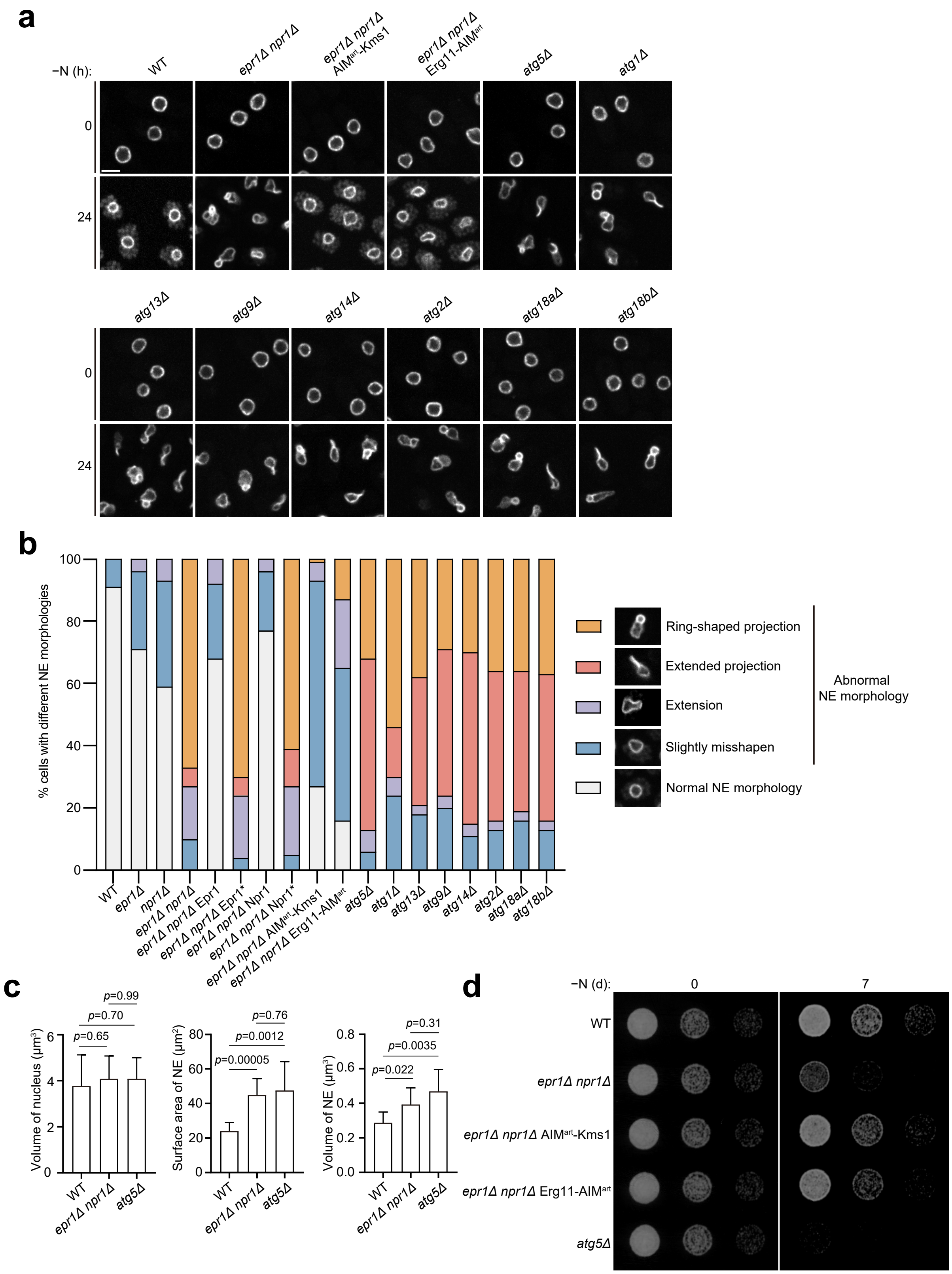

Supplementary Fig. 5: Npr1- and Epr1-mediated nucleophagy maintains nuclear morphology and survival during nitrogen starvation.

(a) Autophagy mutants exhibited NE morphology abnormalities during nitrogen starvation. The NE phenotype in *epr1Δ npr1Δ* was rescued by re-introducing AIM^{art}-Kms1 or Erg11-AIM^{art}. Fluorescence microscopy was used to visualize cells expressing the INM protein mECitrine-Bqt4 before and after 24 h of nitrogen starvation. Bar, 3 μm.
(b) Quantification of the percentages of cells exhibiting normal or various abnormal NE morphologies, as analyzed in Fig. 5a and Supplementary Fig. 5a. Over 600 cells were examined per sample.
(c) Quantification of the nucleus volume, as well as the surface area and volume of the NE, in wild-type, *epr1Δ npr1Δ*, and *atg5Δ* cells. The quantification results are shown as the mean ± s.d. Statistical significance was determined using Student's two-sample *t*-test. The sample sizes for the groups were as follows: wild-type (n=10), *epr1Δ npr1Δ* (n=6), and *atg5Δ* (n=4).
(d) The reduced survival of *epr1Δ npr1Δ* cells was alleviated by re-introducing either AIM^{art}-Kms1 or Erg11-AIM^{art}. Cells subjected to nitrogen starvation for 0 and 7 days were plated in five-fold serial dilutions on YES plates, which were photographed after colony formation.

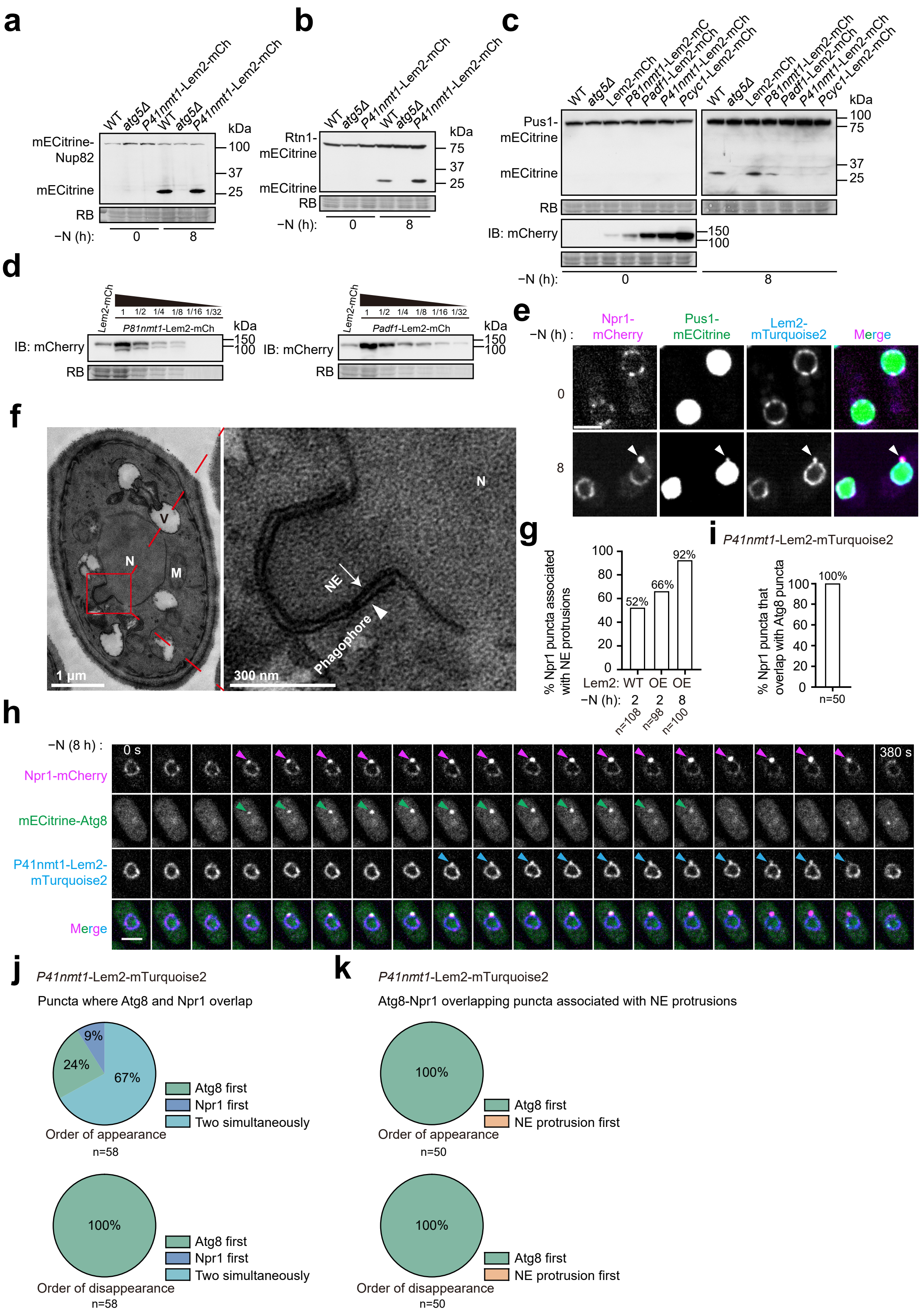

Supplementary Fig. 6: Inhibition of nucleophagy by Lem2 overexpression.

(a) Overexpression of Lem2 does not inhibit the autophagic processing of mECitrine-Nup82.
(b) Overexpression of Lem2 does not inhibit the autophagic processing of Rtn1-mECitrine.
(c) The extent of nucleophagy inhibition depends on the expression level of Lem2. Autophagic processing of Pus1-mECitrine was examined by immunoblotting in cells with endogenously mCherry (mCh)-tagged Lem2 and in cells expressing exogenous Lem2-mCherry from four promoters. The expression strengths of the promoters are as follows: *P81nmt1* < *Padf1* < *P41nmt1* < *Pcyc1.* Lem2-mCh expression levels were analyzed by immunoblotting using an anti-mCherry antibody.
(d) Lem2-mCh levels under *P81nmt1* and *Padf1* were 2-4 times and 8-16 times, respectively, that of the endogenously mCh-tagged Lem2. Log-phase cells were used.
(e) In Lem2-mTurquoise2-overexpressing (OE) cells, Pus1-mECitrine localized to Npr1 puncta-associated NE protrusions after 8 h of nitrogen starvation. Bar, 3 μm.
(f) Electron microscopy analysis of Lem2-OE cells after 6 h of nitrogen starvation showed a phagophore wrapping around an NE protrusion.
(g) Percentages of Npr1 puncta associated with NE protrusions. Each punctum was tracked from appearance to disappearance.
(h) Time-lapse analysis of Lem2-mTurquoise2-OE cells showing Atg8 puncta colocalized with Npr1 puncta and associated NE protrusions, with kinetics similar to WT. Cells co-expressing Npr1-mCherry, mECitrine-Atg8, and Lem2-mTurquoise2 were imaged at 20-s intervals after 8 h of nitrogen starvation. Bar, 3 μm.
(i) Percentages of Npr1 puncta overlapping with Atg8 puncta from time-lapse data. Cells co-expressing Npr1-mCherry, mECitrine-Atg8, and Lem2-mTurquoise2 were analyzed at 20-s intervals after 8 h of nitrogen starvation.
(j) Order of appearance and disappearance of Atg8 and Npr1 at overlapping puncta in Lem2-OE cells.
(k) Order of appearance and disappearance of Atg8 and associated NE protrusions at Atg8/Npr1-overlapping puncta in Lem2-OE cells.

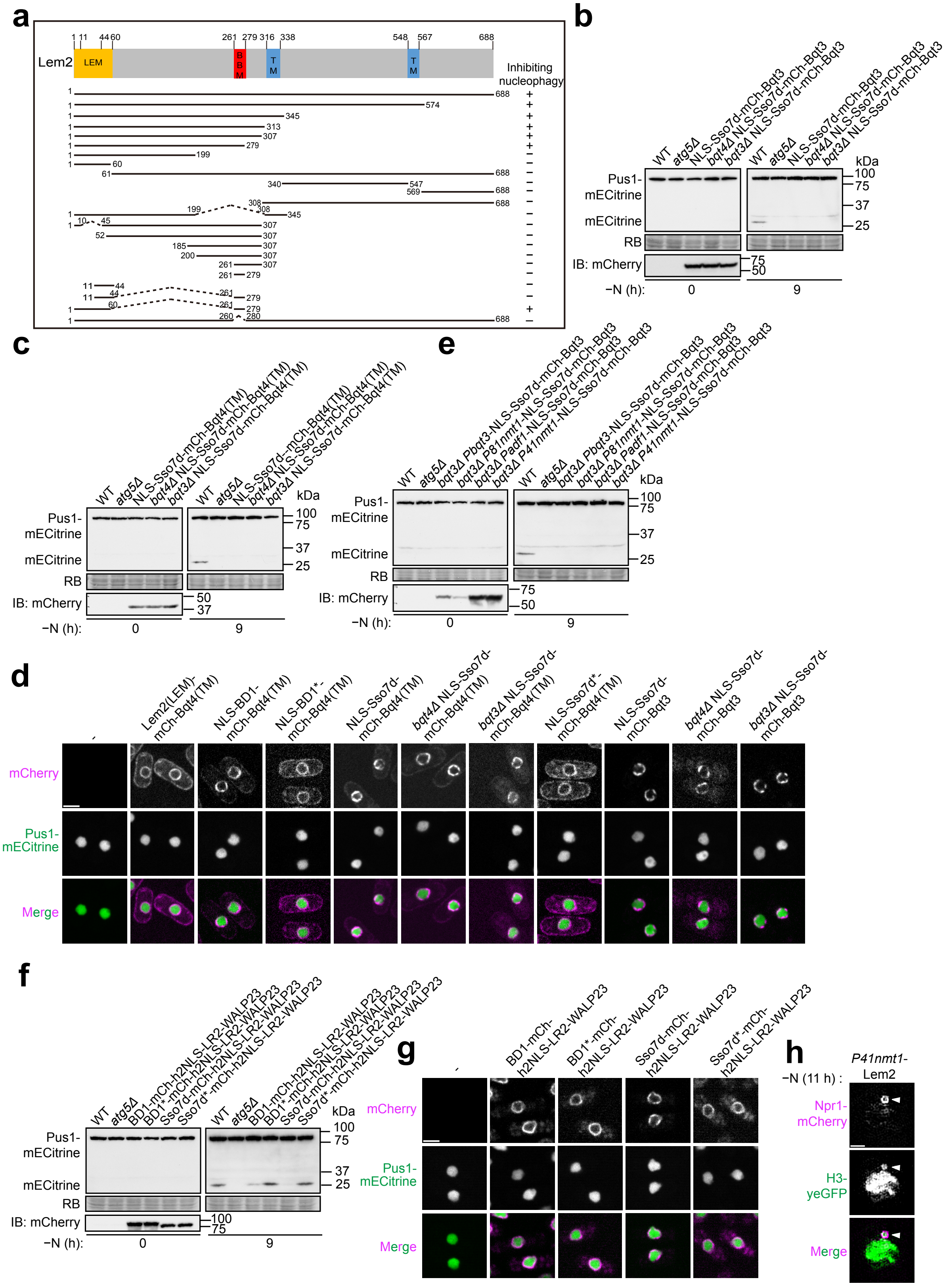

Supplementary Fig. 7: Chromatin-INM tethering inhibits nucleophagy.

(a) Schematic showing the results of truncation and internal deletion analyses of Lem2, which identified the regions required for nucleophagy inhibition. Different forms of Lem2 with truncations or internal deletions were expressed under the *P41nmt1* promoter. Nucleophagy was assessed by monitoring the nitrogen starvation-induced relocalization of Npr1 to the vacuole.

(b) The fusion protein between Sso7d and Bqt3, expressed from the *P41nmt1* promoter, inhibited nucleophagy even when the endogenous *bqt4* or *bqt3* gene was deleted. The fusion protein contains an NLS and mCherry (mCh). Expression levels were analyzed by immunoblotting with an anti-mCherry antibody.

(c) The fusion protein between Sso7d and the C-terminal transmembrane helix (TM) of Bqt4, expressed from the *P41nmt1* promoter, also inhibited nucleophagy in the absence of the endogenous *bqt4* or *bqt3* gene. The fusion protein contains an NLS and mCherry (mCh). Expression levels were analyzed by immunoblotting with an anti-mCherry antibody.

(d) Fusion proteins containing a DNA- or histone-binding domain from Lem2, Bdf1, or Sso7d fused to the C-terminal transmembrane helix (TM) of Bqt4 or full-length Bqt3 localized to the NE. Log-phase cells expressing a fusion protein and the nucleoplasmic protein Pus1-mECitrine were examined by fluorescence microscopy. Bar, 3 μm.

(e) The fusion protein between Sso7d and Bqt3 inhibited nucleophagy even when expressed from the weak *P81nmt1* promoter in *bqt3Δ* cells. The fusion protein contains an NLS and mCherry (mCh). Expression levels were analyzed by immunoblotting with an anti-mCherry antibody.

(f) Targeting BD1 or Sso7d to the INM using h2NLS-LR2-WALP23 inhibited nucleophagy.

(g) Log-phase cells expressing a chromatin-tethering fusion protein and the nucleoplasmic protein Pus1-mECitrine were examined by fluorescence microscopy. Bar, 3 μm.

(h) Super-resolution microscopy showed that histone H3-yeGFP localized within a hollow circular structure where Npr1 accumulated in Lem2-overexpressing cells. Bar, 1 μm.

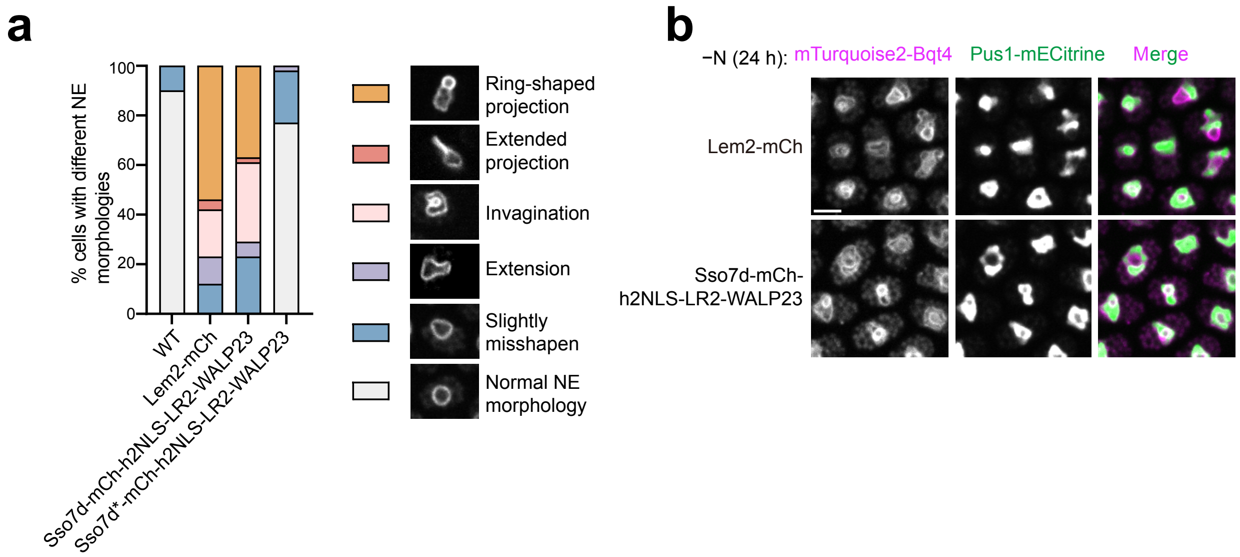

Supplementary Fig. 8: Chromatin-INM tethering resulted in NE morphology abnormalities during nitrogen starvation.

(a) Quantification of the percentages of cells exhibiting normal or various abnormal NE morphologies, as analyzed in Fig. 7f. Over 600 cells were examined per sample.

(b) Pus1-mECitrine was not detected within the ring-like profile of NE projections and was absent from the centers of the invaginations in cells overexpressing Lem2, as well as in cells expressing Sso7d-h2NLS-LR2-WALP23, following 24 h of nitrogen starvation. Bar, 3 µm.
