# Supplementary material for: Nucleophagy is promoted by two autophagy receptors and inhibited by chromatin-nuclear envelope tethering in fission yeast
Source: Nat Commun. 2026 Mar 31;17:4678. doi: 10.1038/s41467-026-71237-x (PMC13201548; doi:10.1038/s41467-026-71237-x)
Supplement: Supplementary file 2 — Description of Additional Supplementary Files [file 41467_2026_71237_MOESM2_ESM.pdf]

Description of Additional Supplementary Files

File Name: Supplementary Data 1
Description: Excel file containing mass spectrometry results of the TurboID experiment.

File Name: Supplementary Data 2
Description: Excel file listing *S. pombe* strains used in this study.

File Name: Supplementary Data 3
Description: Excel file listing plasmids used in this study.

File Name: Supplementary Movie 1
Description: 3D FIB-SEM reconstruction of the NE in a wild-type cell. The NE is shown in blue.

File Name: Supplementary Movie 2
Description: 3D FIB-SEM reconstruction of the NE, NE projection, and vacuoles within the NE projection in an *epr1Δ npr1Δ* cell. The NE is shown in blue. The nuclear projection is shown in yellow. The vacuoles are shown in purple.

File Name: Supplementary Movie 3
Description: 3D FIB-SEM reconstruction of the NE, NE projection, and vacuoles within the NE projection in an *atg5Δ* cell. The NE is shown in blue. The nuclear projection is shown in yellow. The vacuoles are shown in purple.
