# Supplementary material for: Nucleophagy is promoted by two autophagy receptors and inhibited by chromatin-nuclear envelope tethering in fission yeast
Source: Nat Commun. 2026 Mar 31;17:4678. doi: 10.1038/s41467-026-71237-x (PMC13201548; doi:10.1038/s41467-026-71237-x)
Supplement: Supplementary file 9 — Reporting Summary [file 41467_2026_71237_MOESM9_ESM.pdf]

nature portfolio

Corresponding author(s): Li-Lin Du

Last updated by author(s): Nov 6, 2025

Reporting Summary

Nature Portfolio wishes to improve the reproducibility of the work that we publish. This form provides structure for consistency and transparency in reporting. For further information on Nature Portfolio policies, see our Editorial Policies and the Editorial Policy Checklist.

Statistics

For all statistical analyses, confirm that the following items are present in the figure legend, table legend, main text, or Methods section.

n/a	Confirmed	
☐	☒	The exact sample size (*n*) for each experimental group/condition, given as a discrete number and unit of measurement
☐	☒	A statement on whether measurements were taken from distinct samples or whether the same sample was measured repeatedly
☐	☒	The statistical test(s) used AND whether they are one- or two-sided<br>*Only common tests should be described solely by name; describe more complex techniques in the Methods section.*
☒	☐	A description of all covariates tested
☒	☐	A description of any assumptions or corrections, such as tests of normality and adjustment for multiple comparisons
☒	☐	A full description of the statistical parameters including central tendency (e.g. means) or other basic estimates (e.g. regression coefficient) AND variation (e.g. standard deviation) or associated estimates of uncertainty (e.g. confidence intervals)
☐	☒	For null hypothesis testing, the test statistic (e.g. *F*, *t*, *r*) with confidence intervals, effect sizes, degrees of freedom and *P* value noted<br>*Give P values as exact values whenever suitable.*
☒	☐	For Bayesian analysis, information on the choice of priors and Markov chain Monte Carlo settings
☒	☐	For hierarchical and complex designs, identification of the appropriate level for tests and full reporting of outcomes
☒	☐	Estimates of effect sizes (e.g. Cohen's *d*, Pearson's *r*), indicating how they were calculated

Our web collection on statistics for biologists contains articles on many of the points above.

Software and code

Policy information about availability of computer code

Data collection	Fusion (2.3.0.54) on a Dragonfly microscope system were used to acquire fluorescence microscopy images.
Data analysis	Fiji was used to analyze fluorescence microscpy images.<br>CCTOP web server (https://cctop.ttk.hu) was used to predict the transmembrane topology of proteins.<br>AlphaFold2-Multimer was used to predict protein complex structures.<br>Drangonfly Pro (2022.2) was used to analyze FIB-SEM images.

For manuscripts utilizing custom algorithms or software that are central to the research but not yet described in published literature, software must be made available to editors and reviewers. We strongly encourage code deposition in a community repository (e.g. GitHub). See the Nature Portfolio guidelines for submitting code & software for further information.

Data

Policy information about availability of data

All manuscripts must include a data availability statement. This statement should provide the following information, where applicable:

- Accession codes, unique identifiers, or web links for publicly available datasets
- A description of any restrictions on data availability
- For clinical datasets or third party data, please ensure that the statement adheres to our policy

The authors declare that all data supporting the findings of this study are available within the paper and its supplementary information files. Source data are provided with this paper.

Research involving human participants, their data, or biological material

Policy information about studies with human participants or human data. See also policy information about sex, gender (identity/presentation), and sexual orientation and race, ethnicity and racism.

Reporting on sex and gender	N/A
Reporting on race, ethnicity, or other socially relevant groupings	N/A
Population characteristics	N/A
Recruitment	N/A
Ethics oversight	N/A

Note that full information on the approval of the study protocol must also be provided in the manuscript.

Field-specific reporting

Please select the one below that is the best fit for your research. If you are not sure, read the appropriate sections before making your selection.

☒ Life sciences ☐ Behavioural & social sciences ☐ Ecological, evolutionary & environmental sciences

For a reference copy of the document with all sections, see nature.com/documents/nr-reporting-summary-flat.pdf

Life sciences study design

All studies must disclose on these points even when the disclosure is negative.

Sample size	Sample size was not predetermined using statistical methods in this study. Instead, the sample sizes were determined based on community standards for relevant experiments.
Data exclusions	No data were excluded.
Replication	All experiments were repeated independently at least two times. For figures showing results from representative experiments (such as micrographs and blots), we state in figure legends how many times each experiment was repeated independently with similar results.
Randomization	In this study, randomization was not utilized since the experiments were conducted on uniform biological material, specifically yeast cells. As a result, randomization was deemed unnecessary for the purposes of this research.
Blinding	In this study, blinding was not used due to the fact that the experiments were designed and performed by individual investigators who were aware of the identities of the analyzed samples. Blinding is not typically employed in this field of research.

Reporting for specific materials, systems and methods

We require information from authors about some types of materials, experimental systems and methods used in many studies. Here, indicate whether each material, system or method listed is relevant to your study. If you are not sure if a list item applies to your research, read the appropriate section before selecting a response.

Materials & experimental systems

n/a	Involved in the study
☐	☒ Antibodies
☒	☐ Eukaryotic cell lines
☒	☐ Palaeontology and archaeology
☒	☐ Animals and other organisms
☒	☐ Clinical data
☒	☐ Dual use research of concern
☒	☐ Plants

Methods

n/a	Involved in the study
☒	☐ ChIP-seq
☒	☐ Flow cytometry
☒	☐ MRI-based neuroimaging

Antibodies

Antibodies used

Primary and secondary antibodies used in this study are all from commercial sources. They include:
1. Mouse monoclonal anti-GFP, Roche, Cat#11814460001
2. Rabbit polyclonal anti-mCherry, ThermoFisher, Cat#PA5-34974
3. Goat anti-Mouse IgG, Sigma Aldrich, Cat#A4416
4. Goat anti-Rabbit IgG, Sigma Aldrich, Cat#A6154

Validation

Validation information for the primary antibodies used in this study is shown below.
1. Mouse monoclonal anti-GFP, Roche, Cat#11814460001
https://www.sigmaaldrich.cn/CN/en/product/roche/11814460001
Google Scholar search showed that this antibody has been used in thousands of publications.
One of the earliest publications using this antibody is Arts et al. Nat Genet 2007 (PMID: 17558407), which used this antibody for immunoblotting.
2. Rabbit polyclonal anti-mCherry, ThermoFisher, Cat#PA5-34974
https://www.thermofisher.cn/cn/zh/antibody/product/mCherry-Antibody-Polyclonal/PA5-34974
The above web page lists 64 publications using this antibody. One of the earliest publications using this antibody is Dejanovic et al. EMBO Mol Med. 2015 (PMID: 26613940), which used this antibody for immunoblotting.
